# Screening nested-PCR primer for *‘Candidatus Liberibacter asiaticus’* associated with citrus Huanglongbing and application in Hunan, China

**DOI:** 10.1371/journal.pone.0212020

**Published:** 2019-02-22

**Authors:** Yanyun Hong, Yongyang Luo, Jianglan Yi, Ling He, Liangying Dai, Tuyong Yi

**Affiliations:** 1 Hunan Provincial Key Laboratory for Biology and Control of Plant Pests, College of Plant Protection, Hunan Agricultural University, Changsha, Hunan province, China; 2 College of life Science and Technology, Beijing University of Chemical Technology, Beijing, China; Fujian Agriculture and Forestry University, CHINA

## Abstract

Citrus Huanglongbing (HLB) is one of the most devastating citrus diseases worldwide. Sensitive and accurate assays are vital for efficient prevention of the spread of HLB-associated “*Candidatus Liberibacter spp*”. “*Candidatus Liberibacter spp*” that infect Citrus includes “*Candidatus Liberibacter asiaticus*” (Las), “*Candidatus Liberibacter africanus*” (Laf) and “*Candidatus Liberibacter americanus*” (Lam). Of them, Las is the most widespread species. In this study, a set of nested PCR primer pairs were screened to diagnose Las, and the nested PCR method greatly enhanced the sensitivity to detect Las up to 10 times and 100 times compared to qPCR and conventional PCR, respectively. Totally, 1112 samples from 5 different citrus cultivars in 39 different counties and cities were assayed by nested PCR. The results show that 384 samples were HLB-infected; the highest positive detection rate was 79.7% from the lopsided fruit samples, and the lowest positive detection rate was 16.3% from the apical dieback samples. The results indicate that the designed nested PCR primer pairs can detect Las from different symptomatic tissues, different citrus cultivars and different geographic regions. The set of nested PCR primers designed in the present study will provide a very useful supplementation to the current approaches for Las detection.

## Introduction

Citrus Huanglongbing (HLB), also known as citrus greening, has been one of the most devastating diseases to threaten the citrus industry in Asia, Africa and America [1]. The citrus trees acutely infected by HLB show yellow shoots, foliar blotchy mottle that may be similar to the symptom of zinc deficiency, vein corking that may be identical to the symptom by the infection of *Citrus Tristeza* virus, poor flowering and stunting [2]. The citrus trees chronically infected with HLB are sparsely foliated, display extensive twig or limb die-back and will eventually die within three to five years [3, 4]. The yield of HLB-infected citrus is reduced by 30% to 100%, and the fruit quality is degraded [1, 2, 5]. It was reported that HLB may reduce Texas citrus production by 20% ($140 million) after 2 years and up to 60% after 5 years of infection [1]. HLB is caused by ‘*Candidatus Liberibacter spp*’, which are gram-negative, unculturable, phloem-limited organisms that belong to α subdivision of the Proteobacteria. HLB-associated *Candidatus Liberibacter spp* include “*Candidatus Liberibacter asiaticus*” (Las), “*Candidatus Liberibacter africanus*” (Laf) and “*Candidatus Liberibacter americanus*” (Lam) [3]. Las is the most widespread species and is responsible for the main increasing economic losses [6, 7]. HLB was transmitted by budding, dodder, grafting and the psyllid vectors. HLB is spread very fast that the spread distance could reach 193 km (120 miles) per year [8, 9]. Unfortunately, currently, there is no effective curative treatment to **c**ontrol this disease, and no cultivars are resistant to this pathogen. Controlling the insect vector and removal of the infected trees are the most general control measures in HLB management. Therefore, sensitive and accurate diagnosis is a prerequisite to research and manage HLB.

Due to that HLB bacteria could not be cultured, Koch's postulates on HLB were not performed [10]. Meanwhile, HLB-infected citrus lacking specific symptoms or that was asymptomatic during the incubation period resulted in some false-negative diagnoses based on visual symptoms [4]. Currently, many molecular detection assays based on PCR, including conventional PCR [11, 12], SSR [13, 14], droplet digital PCR [15], LAMP[16, 17], immune capture-PCR[18], qPCR [4, 19] and nested PCR [20], have been used to detect HLB-associated bacteria. Nested PCR, whose products of the first round of PCR are diluted and used as the template for the second round of amplification, has been proven to have higher sensitivity than other molecular detection assays in diagnosing diseases[21–23] such as HLB[24, 25]. Thus, the nested PCR was used in this study.

Many types of genomic loci have been used as molecular markers in the detection of HLB. For example, the 16S rRNA gene rplKAJL-rpoBC cluster region, intergenic 16S/23S rRNA gene spacer region, bacteriophage-type DNA polymerase region, 18S rRNA gene and the outer membrane protein (OMP) gene were widely used in the molecular detection of HLB [15, 25–27]. However, it was noticeable that molecular detection assays were subject to false negative results owing to uneven distribution of the HLB bacteria in citrus and even among cells within discrete tissues, yielding both positive and negative samples from the same citrus tree. Moreover, the same HLB bacteria species had significant differences among various geographic locations, and the same HLB bacteria species had no significant difference among different citrus cultivars [27, 28]. Therefore, it is important to choose an appropriate assay method and a proper genomic locus to detect the suspicious HLB samples from a particularly geographical location. The *OMP* (outer member protein) gene of *Candidatus Liberibacter asiaticus*, with 2,346 bp of nucleotides, was first sequenced in 2005[29]. The three-dimensional structures of OMP were highly conserved, and the nucleotide sequences of the *OMP* gene showed very high similarity among isolates (99%) and high species specificity (99%) [29, 30]. In this study, *OMPs* and nested PCR were used as target genes and the detection assay method, respectively, to detect HLB- suspicious samples collected from Hunan province in China.

## Materials and methods

### Ethics statement

The research complied with protocols approved by the plant protection and plant inspection station of Hunan Province, China, and abided by the legal requirements of China. The research was conducted according to plant protection regulations.

### Sample sources and DNA preparation

**Fig 1:**
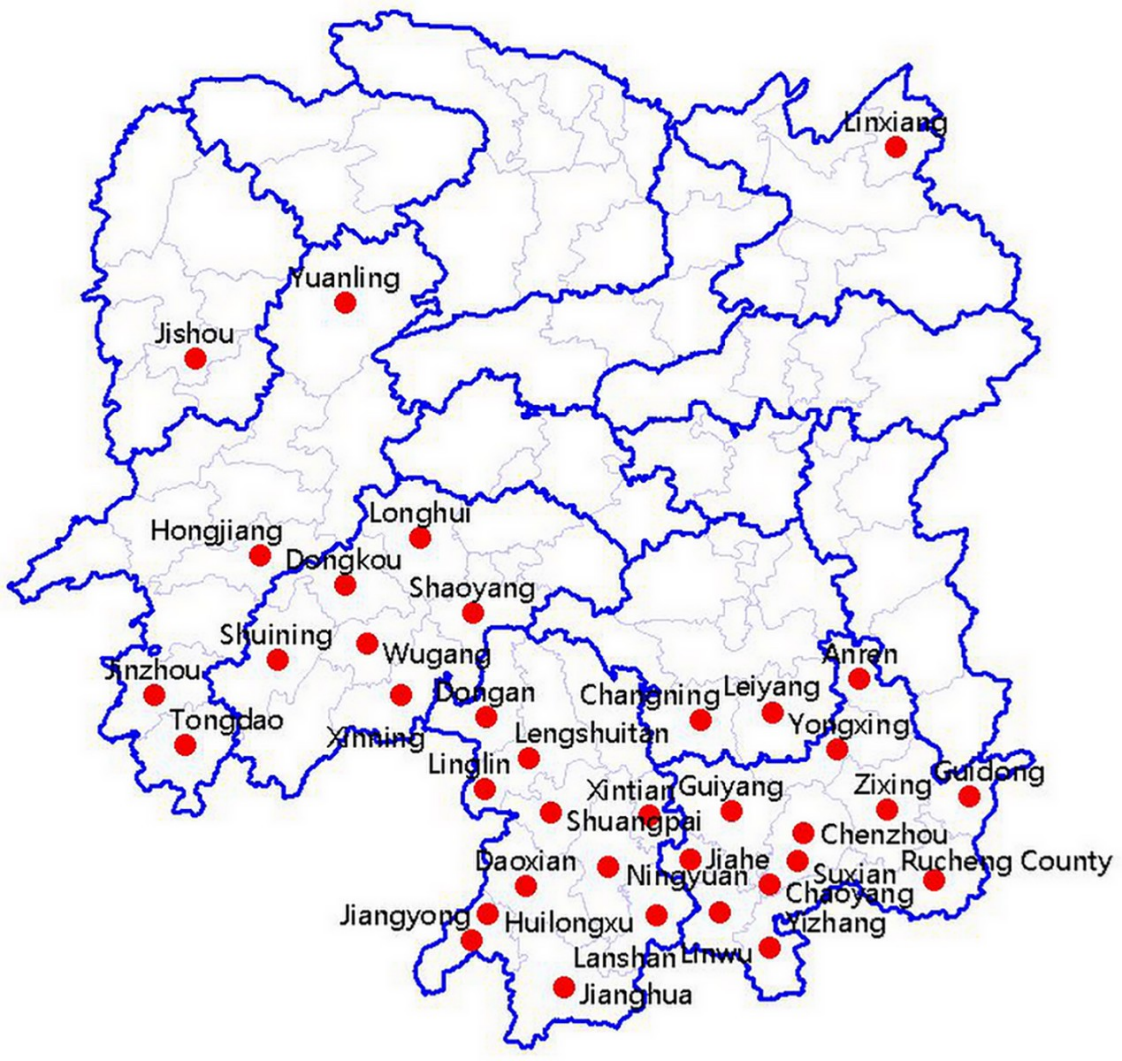
Map of Hunan Province, China with red dots indicating locations where the studied samples were collected.

From 2014 to 2017, 1112 citrus tissue samples, including 154 lopsided fruits in green in color and 958 leaf samples that 422 showed blotchy leaves citrus, 389 showed yellow shoots, 147 showed apical dieback, were collected from 5 different citrus cultivars in 39 different counties and cities that covered almost all citrus cultivars and planting areas in Hunan province, China(East Longitude:109°68′-113°96′;North Latitude: 24°97′-29°48′) (Table 1 and Fig 1). All samples were collected and sent to us by concern counties plant protection station (S1 Table).Leaves and fruits were washed with running tap water and blotted dry with paper towels. The midribs and fruit peels were excised. Then, 100 mg of each sample were ground in liquid nitrogen, and DNA was extracted using the CTAB method as previously described [31]. The extracted DNA was dissolved in 50 μl of TE buffer. The quality and concentration of DNA were checked by a NanoDrop ND-2000 (NanoDrop Technologies Inc., Wilmington, DE, USA)[32,33].

**Table 1:** Information of the collected samples including the locations, symptoms and numbers.

No.	Location	GPS	Symptom of Samples			
			Botchy leaf	Yellow shoot	Apical dieback	Lopsided fruit
1	Chenzhou City	113°E25°79′N	3	4	0	0
2	Anren County	113°27′E26°71′N	18	10	16	6
3	Yizhang County	113°96′E25°41′N	12	10	2	3
4	Yongxing County	113°11′E26°13′N	7	9	0	2
5	Zixing County	113°39′E25°95′N	7	9	1	0
6	Guidong County	113°91′E25°08′N	7	1	0	5
7	Guiyang County	112°72′E25°73′N	22	23	7	6
8	Jiahe County	112°35′E25°56′N	9	5	5	3
9	Linwu County	112°55′E25°27′N	13	5	1	3
10	Rucheng County	113°68′E25°54′N	6	4	1	13
11	Chaoyang District	113°02′E25°79′N	3	1	0	1
12	Suxian District	113°03′E25°49′N	10	14	0	1
13	Changling City	112°39′E26°38′N	1	0	0	0
14	Leiyang City	112°84′E26°41′N	6	6	1	0
15	Dao County	111°57′E25°52′N	14	13	4	1
16	Dongan County	111°28′E26°41′N	11	7	6	6
17	Jianghua County	111°79′E24°97′N	66	57	12	17
18	Jiangyong County	111°33′E25°41′N	92	81	32	30
19	Xintian County	112°21′E25°91′N	5	1	0	0
20	Lanshan County	112°16′E25°37′N	5	2	0	0
21	Linshuitan City	111°29′E26°15′N	0	0	3	2
22	Linglin District	111°63′E26°22′N	2	6	0	0
23	Ningyuan County	111°95′E25°6′N	7	10	1	2
24	Shuanpai County	111°64′E25°96′N	35	30	6	7
25	Huilongxu	111°24′E25°42′N	6	10	5	6
26	Dongkou County	110°57′E27°06′N	3	5	6	3
27	Wugang County	110°61′E26°73′N	0	0	6	2
28	Longhui County	111°04′E27°13′N	5	3	2	0
29	Xinning County	110°84′E26°44′N	23	37	14	16
30	Shaoyang County	111°5′E27°22′N	1	2	0	3
31	Suining County	110°14′E25°59′N	3	3	2	6
32	Jieshou City	109°71′E28°3′N	2	7	0	4
33	Hongjiang City	109°96′E27°1′N	2	2	1	0
34	Jinzhou County	109°68′E26°57′N	6	2	0	0
35	Tongdaozizhizhou County	109°77′E26°16′N	5	2	3	2
36	Yuanlin County	110°39′E28°46′N	2	3	2	0
37	Ningxiang County	113°42′E29°48′N	1	2	2	1
38	unknown		0	0	2	2
39	unknown		2	3	4	1
Total			422	389	147	154

### Primers design

Two sets of primers were designed for the nested PCR to amplify the conserved region of BamA that encodes OMP assembly factor and is annotated as a single copy in the Las genome (Accession No: JQ928882.1) [34–36]. The optimum inner and qPCR primers were designed using Beacon Design Software v7.0 (Premier Biosoft International, CA, USA) with the following criteria: GC %≥40–50, Tm = 60 ±2°C, and primer length = 18–22 bp. To ensure amplification efficiency, among the designed primers that had the least possibility in forming a hairpin, self/cross dimer structures were selected for further validation. For designing the outer primers, the same criteria were applied, except that a longer amplicon size (i.e., 1000–1500 bp) was designed. At the same time, the conventional PCR primer OI1 /OI2 and S3/S4 were obtained from previous study [12]. Three sets of nested PCR primers were validated (F1/B1 and F3/B3; F2/B2 and F3/B3; OI1/OI2 and S3/S4). To identify these pathogenic microorganisms used in validation the specificity of designed nested-PCR primers indeed existed in those DNA samples, Xac1/Xac2 (Accession No: KY849808.1), Spir1/Spir2 (Accession No: KT377378.1), Citrus actin(Accession No: XM_006464503.3) and Potato actin(Accession No: X55749.1) were designed according to the same criteria. All the primers were synthesized by Sangon Biotech (Shanghai) Co., Ltd. All the primers information are displayed (Table 2).

**Table 2:** Information of primers used in this study.

Name	Forward primer(5'-3')	Reverse primer(5'-3')	Tm	Produc tion(bp)	Target gene
			(°C)		
F1/B1 F2/B2	GGTTATGCTGCCGTTAAAGTGTC GGTTATGCTGCCGTTAAAGTGTC	AACCAGCCCTTTCAGGAACAAG AACCAGCCCTTTCAGGAACAAG	58 58	1318 1245	OMP OMP
F3/B3	TCTGAGGGTGAGCGTAAAACAACTG	TTGGGAAATAGAATGGCTGCTGAAT	62	447	OMP
OI1/OI2	GCGCGTATCCAATACGAGCGGCA	GCCTCGCGACTTCGCAACCCAT	62	1160	16SrDNA
S3/S4	GTAAACGATGAGTGCTAGCTGT	CTATAAAGTACCCAACATCTAGGT	59	359	16SrDNA
qPCR	GCC ACGTAA AGG CAT GTT GA	GCT CGA GATCCA ATC CGA TG	60	116	OMP
OA1/OI2c	GGGGTAAATGCCTACCAAGG	GCACCGTCTTCAAGCAAAAC	56	1160	16SrDNA
ZFC/OI2c	GTTGTAAAGCTCTTTCGCCG	GCACCGTCTTCAAGCAAAAC	57	893	16SrDNA
Xac1/Xac2	CGCCATCCCCACCACCACCACGAC	AACCGCTCAATGCCATCCACTTCA	55	582	ATP synthase et
Spir1/Spir2	GGAAGTAAAAGTCGTAACAAGG	GCTGCGTTCTTCATCGATGC	55	529	transmembrane adhesion
Citrus actin	GTATGCCACGTCGCATTCCAGA	GCCAAAACTGCTAAGGGCATTC	55	112	Actin
Potato actin	GCTCTCAAAGATCGGTTTGACGG	GCTGCCACGAACGTTACCTTC-	53	287	Actin

### Validation the specificity of designed nested-PCR primers

To validate the nest-PCR primer specificity, we firstly BLAST all primers against citrus and Asian citrus psyllid (ACP) sequences. To ensure that the designed primers were specific for Las, the specificities of the outer and inner primers(F1/B1 and F3/B3) were evaluated by nested-PCR using DNAs extracted from other citrus-related pathogens, such as *Xanthomonas citri subsp* and *Spiroplasma citri* preserved in our laboratory. Moreover, three DNAs of Laf, Lam and “*Ca*. *L*. *solanacearum*” generous gifted by the “Citrus Research Institute Chinese Academy of Agricultural Science” were also used for specificity evaluation. To identify these pathogens in those DNA samples, special primers OI1 + OA1+ OI2c, described by Jagoueix et al (1996), were used to amplify Laf and Lam; primers ZCf/OI_2_c were used to amplify *Ca*. *L*. *solanacearum*, primers Xac1/Xac2 were used to amplify *Xanthomonas citri subsp;* primers Spir1/Spir2 were used to amplify *Spiroplasma citri*. At the same time, Citrus and potato actin gene as plant control were amplified. Objective genes were confirmed by electrophoresis in 1.2% agarose gels and sequencing PCR products and BLAST them online (Table 2 and S1 Appendix). To confirm whether the locus sequence is conserved and shared among *“Candidatus Liberibacter asiaticus”* isolates, seven DNAs of Las, which were collected in Hunan, Guangdong, Fujian, Jiangxi, Yunnan, Sichuan and Guangxi provinces and stored at“Citrus Research Institute of Hunan”, were tested using the nested PCR primers(F1/B1 and F3/B3) in this study. PCR products were separated by electrophoresis in 1.2% agarose gels and detected after staining with ethidium bromide.

### Validation of the sensitivity of nested-PCR

**Fig 2:**
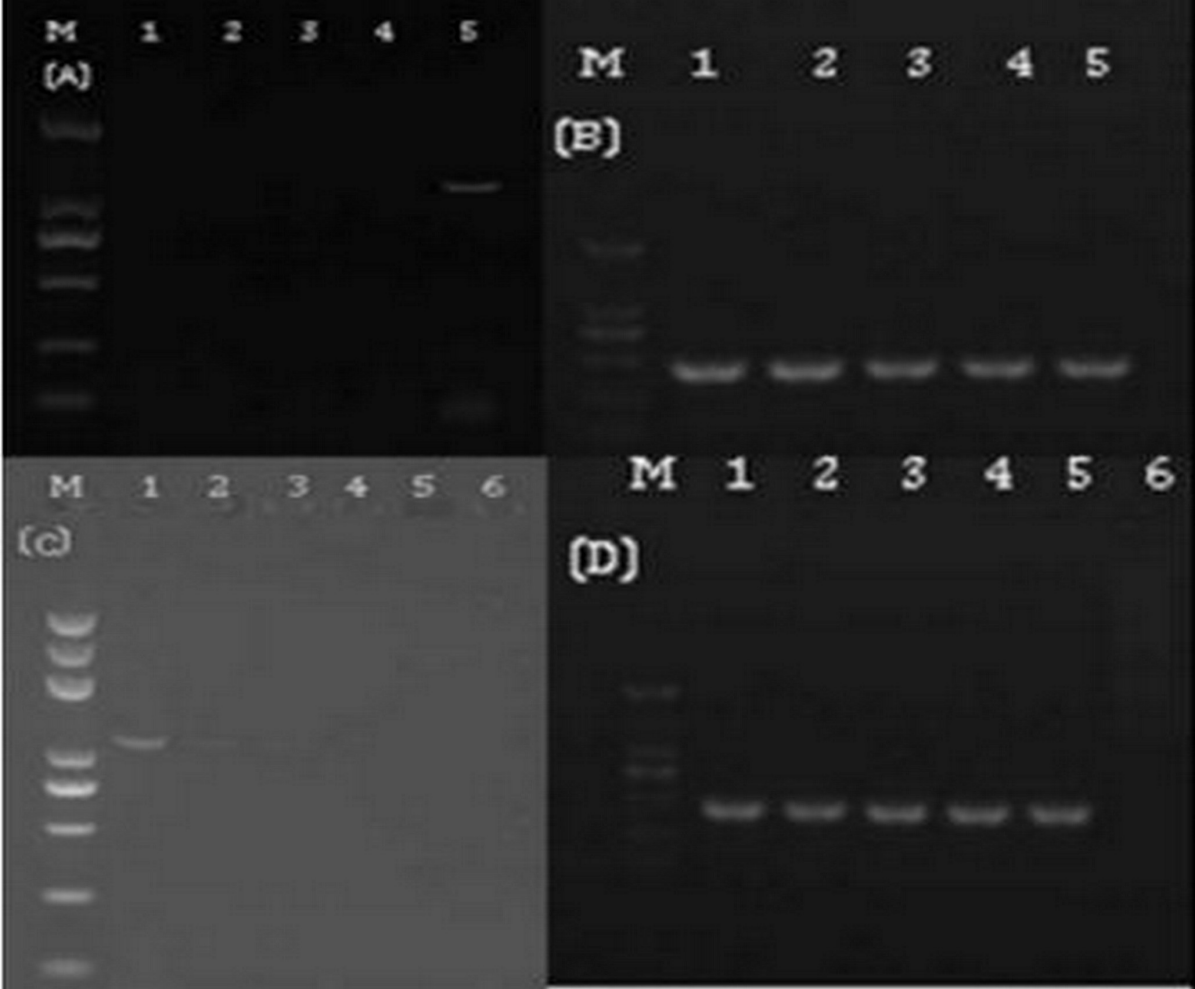
Electrophoretic comparisons of PCR products from conventional and nested PCR and dilution series analyses. A- conventional PCR of five different samples; B-nested PCR of the same five samples as shown in A; C-conventional PCR of 10^5^ fold dilution series of PCR product shown in Fig 2A-lane 5 and D-nested PCR of dilution series of PCR product shown in Fig 2-B-lane 5. (Marker: 2000bp, 1000bp, 750bp, 500bp, 250bp, 100bp).

Usually, the determination of primer sensitivity was based on different templates or different concentrations of same template. Firstly, five suspected samples were amplified by nested PCR primer pairs (F1/B1 and F3/B3) and conventional PCR primer (OI1/OI2). Then we detected the concentration of the conventional PCR positive product and nested PCR positive product from Fig 2A or 2B lane 5 by a NanoDrop ND-2000, and adjusted to 100 ng/μl as the dilution template. The PCR product was serially diluted in a range of 1, 10, 10^2^, 10^3^, 10^4^ and 10^5^, which represented 10^5^ to 1 pg/μl Las DNA, and served as the templates to evaluate the sensitivity among nested PCR, conventional PCR and qPCR. The PCR product from 2A line 5 was diluted as the template for conventional PCR (Fig 2C) and the PCR product from 6B line 5 was diluted as the templates for the nested PCR (Fig 2D) and qPCR. To evaluate the amplification efficiency of qPCR, Las DNA (Fig 2B) serially diluted in a range of 10, 10^2^, 10^3^, 10^4^ and 10^5^ served as the templates. The nested PCR mixture (20 μl) was prepared using 2×Easy Taq PCR SuperMix (Transgen Biotech, Beijing, China), and amplification was proceeded using the following parameters: 94°C for 5 min followed by 25 cycles at 94°C for 30 s, 58°C for 30 s and 72°C for 70 s for the first round of PCR and 35 cycles at 94°C for 30 s, 62°C for 30 s and 72°C for 30 s for the second round of PCR. The qPCR mixture (20 μl) was prepared using Trans Start Top Green qPCR SuperMix (Transgen Biotech, Beijing, China), and amplification was proceeded using the following parameters: 94°C for 30 s and followed by 40 cycles at 94°C for 5 s and 60°C for 30 s, and followed by a melt curve (60°C to 90°C, 0.3°Cs-1). At the same time, 2×Easy Taq PCR SuperMix was used in conventional PCR assays. All PCR mixtures (20 μl) included 1 μl of DNA and 20 pmol of primer pairs. Conventional PCR amplification was performed according to early literature [12]. DNA-free H2O citrus was amplified as negative controls. Nested PCR and conventional PCR products were separated by electrophoresis in 1.2% agarose gels and visualized after staining with ethidium bromide. When the Ct value was less than 36, the DNA sample was identified as HLB-infected.

## Results

### Primers selected for nested-PCR detection system

**Fig 3:**
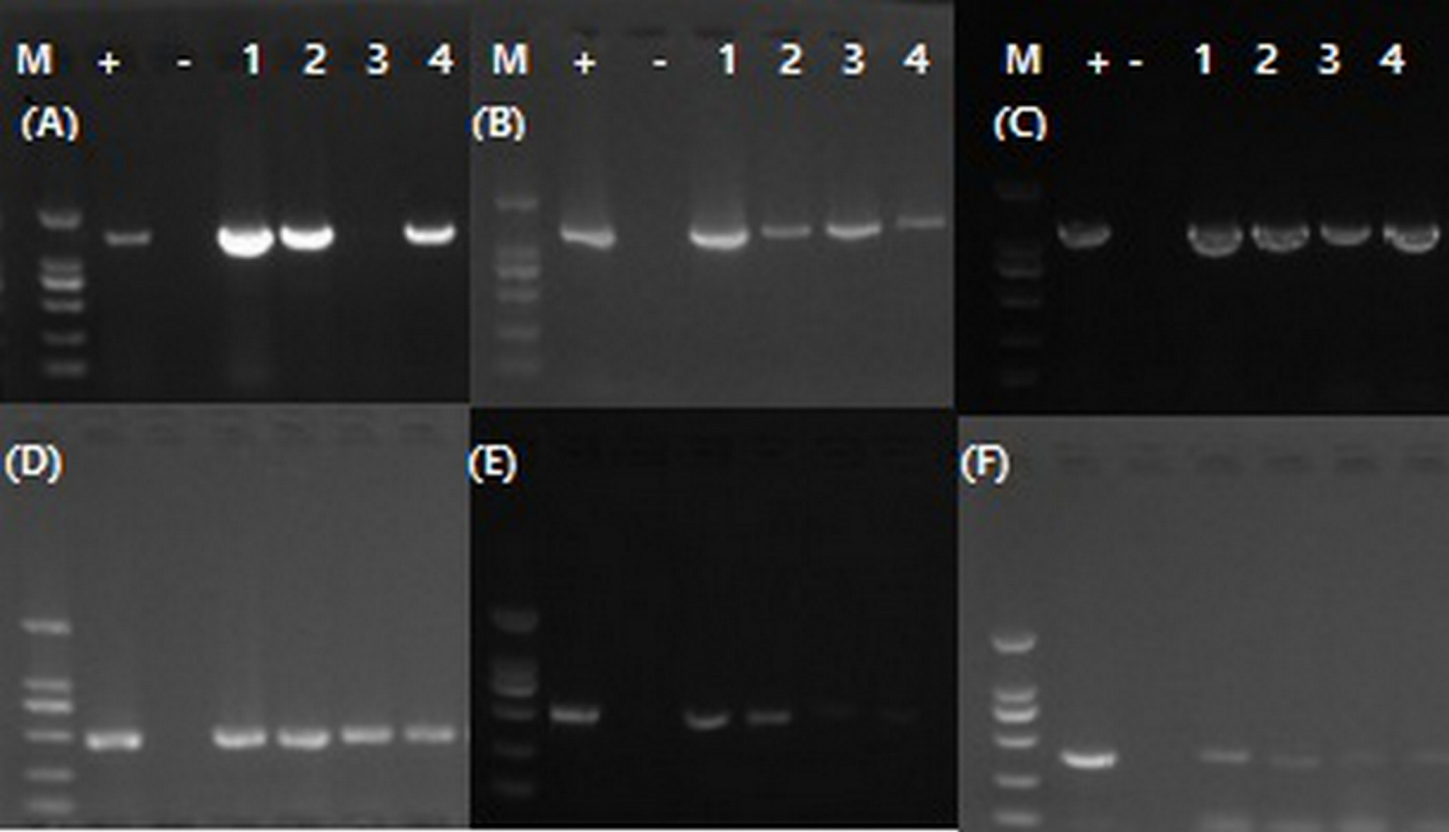
The electrophoresis results of products of three pairs of nest-PCR using four DNA samples, which included two lopsided fruits and one yellow shoot and one blotchy leaf. +, DNA of Las; -, Negative controls; 1 and 2, lopsided fruits; 3, yellow shoot; 4, blotchy leaf. Each picture was a pair of primer. A, F1/B1; B, F2/B2; C, OI1/OI2; D, F3/B3; E, F3/B3; F, S3/S4. (Marker: 2000bp, 1000bp, 750bp, 500bp, 250bp, 100bp).

Three sets of nested PCR primers produced the target products without primer dimers. However, the primers F1/B1 and F3/B3 gave the strongest bands compared to the other two sets of primers according to electrophoresis results (Fig 3). Therefore, the primers F1/B1 and F3/B3 were selected for this study (Table 2). The amplification products are 1318 bp in length for the outer primers and 447 bp in length for the inner primers.

### Validation of nested-PCR primer specificity

**Fig 4:**
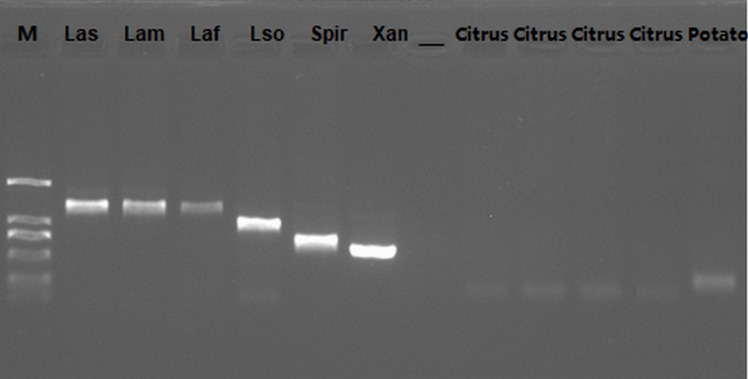
The electrophoresis results on pathogens and plants control. 1, Las; 2, Laf; 3, Lam; 4, *Ca*. *L*. *solanacearum; 5*, *Xanthomonas citri subsp; 6*,*Spiroplasma citri;* 7, negative control; 8–11 citrus controls corresponding to Laf, Lam, *Xanthomonas citri subsp* and *Spiroplasma citri*; 12 potato control corresponding to *Ca*. *L*. *solanacearum* (Marker: 2000bp, 1000bp, 750bp, 500bp, 250bp, 100bp).

**Fig 5:**
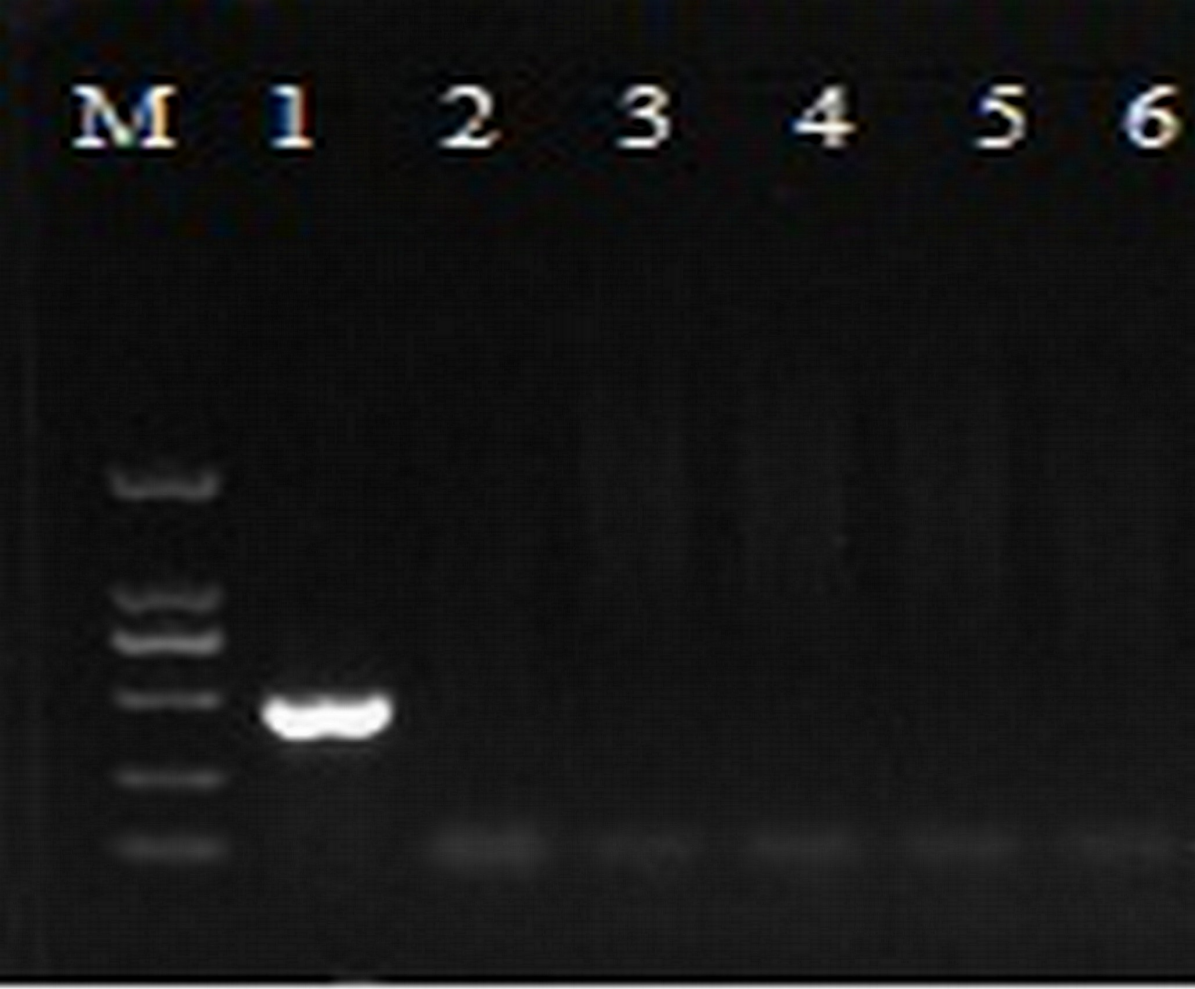
The electrophoresis results on nested PCR primer specificity. 1, Las; 2, Lam; 3,Laf; 4, *Ca*. *L*. *solanacearum; 5*, *Xanthomonas citri subsp; 6*,*Spiroplasma citri*.(Marker: 2000bp, 1000bp, 750bp, 500bp, 250bp, 100bp).

**Fig 6:**
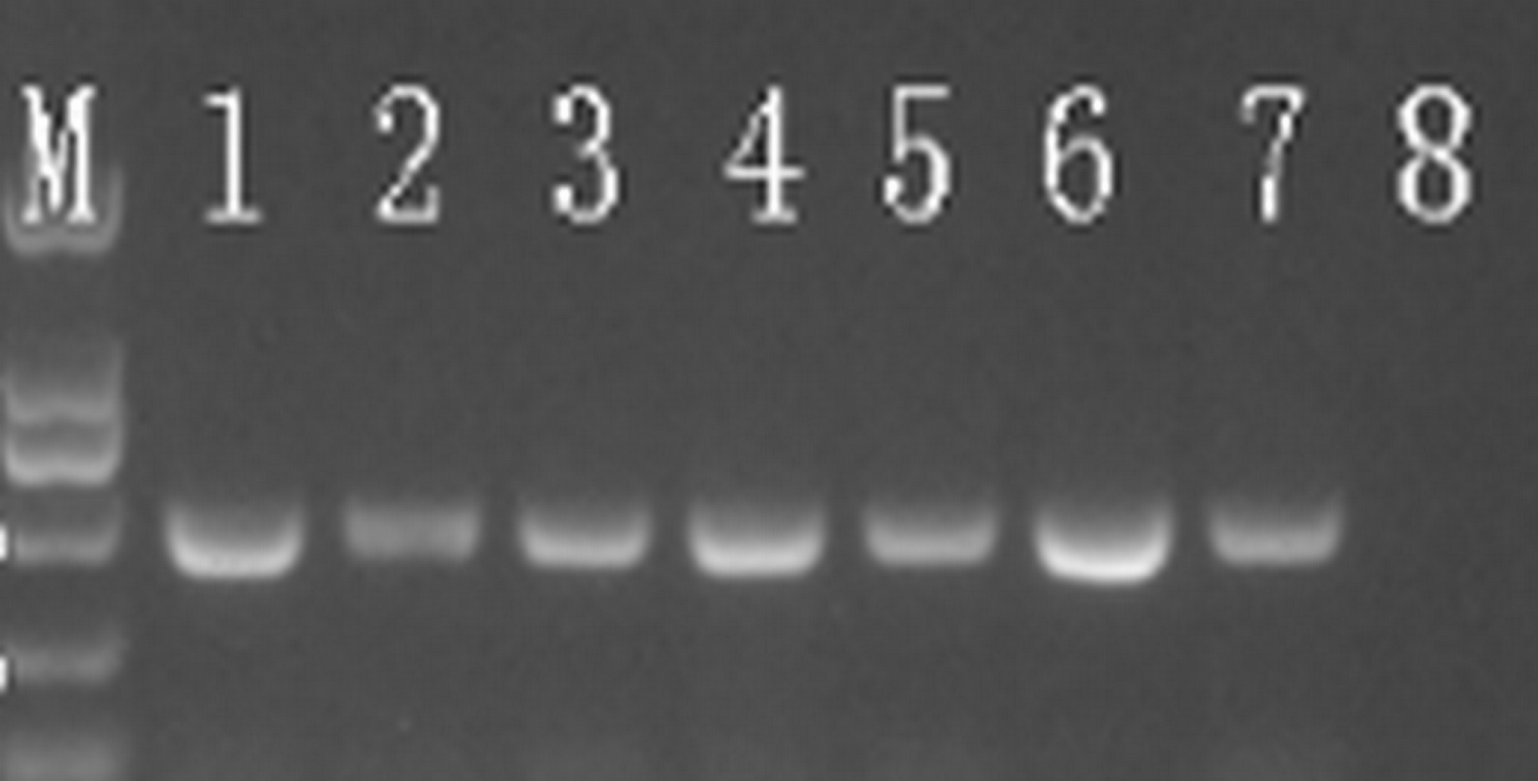
The electrophoresis results of nested PCR pairs (F1/B1 and F3/B3) from different geographic Las strains. 1, Hunan;2,Guangxi;3,Fujian;4,Yunnan;5,Guangdong; 6,Sichuan;7,Jiangxi; 8, negative control (Marker: 2000bp, 1000bp, 750bp, 500bp, 250bp, 100bp).

To validate the nest-PCR primer specificity, we firstly BLAST all primers against citrus and Asian citrus psyllid (ACP) sequences, the results showed all primers had regions with 100% identity to some citrus gene sequences and ACP gene sequences, but showed very low coverage (52–75%) to citrus and ACP gene sequences. For example, F1 was 68% coverage to Citrus sinensis methionyl-tRNA formyltransferase and B1 was 60% coverage to Citrus sinensis fasciclin-like arabinogalactan protein 17, F1 was 52% coverage to citrus psyllid sarcoplasmic reticulum histidine-rich calcium-binding protein-like and B1 was 59% coverage to citrus vacuolar protein sorting-associated protein 37A-like. Then we sequenced the products of the nested-PCR, and BLAST these gene fragments against Citrus sequences and ACP sequences and microbial sequences. The results showed no other homologous sequences except Las—related genes sequence were found, suggesting the set of primers was specific to Las(S2 Appendix). According to the results of electrophoresis and BLAST online, all pathogenic bacteria/fungi (Las, Laf, Lam, *Ca*. *L*. *solanacearum*, *Xanthomonas citri subsp*, *Spiroplasma citri*) indeed existed in DNA samples (Fig 4 and S1 Appendix).However, the negative results of several other pathogens further confirmed the specificity of nested-PCR primer pairs (Fig 5). The results of Las isolates from different geographical regions, which were determined by F1/B1 and F3/B3, showed positive, confirming that the sequence locus was conserved and shared among these Las isolates (Fig 6).Therefore, the selective primers are species-specific and highly conserved.

### Comparison of the sensitivity among different detection methods

According to the results of electrophoresis, with conventional PCR only the sample in lane 5 was detected as Las-infected (Fig 2A); however, with nested PCR all 5 samples were detected as Las infected (Fig 2B). Analysis of the dilution series of the DNA from lane 5 showed that nested PCR (Fig 2D) detected much lower template DNA concentrations than conventional PCR (Fig 2C). The results indicted that the lowest concentration detected by conventional PCR was 1×10^3^ pg/μl, and the lowest concentration detected by nested PCR was 1× 10 pg/μl(Fig 2). When the qPCR CT value was 35, the template DNA concentration was 1×10^2^pg/μl. Accordingly, the primer sensitivity was inversely correlated with the template concentration; the detection system sensitivity order was nested PCR>qPCR> conventional PCR, meaning that the nested PCR was 10 times more sensitive than that of qPCR and 100 times more sensitive that of conventional PCR. The standard curve in this study had an average slope value of -3.31. The amplification efficiency (AE) of qPCR was therefore estimated to be approximately 0.99 based on the equation AE = [10^−1/slope^−1].

### Detection of HLB in the field samples by nested-PCR

In the study, 1111 field samples were assayed by nested PCR. Totally, 384 samples, including 140 blotchy leaves, 98 yellow shoots, 24 apical dieback samples and 122 lopsided fruits, were diagnosed as HLB-infected. The rate of HLB infection was 34.5%. However, the highest positive detection rate was 79.7% in lopsided fruits, and the lowest positive detection rate was 16.3% in apical dieback samples. There were 36.5%, 25.5%, 6.3% and 31.8% positives among the trees diagnosed based on blotchy leaves, yellow shoots, apical dieback samples and lopsided fruits, respectively (Table 3)

**Table 3:** The results from nested PCR pairs (F1/B1 and F3/B3).

No.	Location	HLB^+^			
		Blotchy leaf	Yellow shoot	Apical dieback	Lopsided fruit
1	Chenzhou City	0	1	0	0
2	Anren County	2	0	0	6
3	Yizhang County	2	6	1	3
4	Yongxing County	3	1	0	2
5	Zixing County	1	5	0	0
6	Guidong County	3	0	0	5
7	Guiyang County	9	4	2	6
8	Jiahe County	3	4	0	2
9	Linwu County	6	3	0	3
10	Rucheng County	3	4	1	12
11	Chaoyang District	1	0	1	1
12	Suxian District	2	0	0	1
13	Changling City	0	0	0	0
14	Leiyang City	0	0	0	0
15	Dao County	10	3	0	1
16	Dongan County	6	2	0	5
17	Jianghua County	26	16	7	15
18	Jiangyong County	25	25	5	18
19	Xintian County	2	1	0	0
20	Lanshan County	2	0	0	0
21	Linshuitan City	0	1	0	2
22	Linglin District	1	1	2	0
23	Ningyuan County	0	2	0	2
24	Shuanpai County	11	10	0	6
25	Huilongxu	7	3	0	4
26	Dongkou County	1	2	1	1
27	Wugang County	3	1	0	1
28	Longhui County	1	0	0	0
29	Xinning County	2	1	0	10
30	Shaoyang County	2	0	0	3
31	Suining County	1	0	2	5
32	Jieshou City	0	0	0	4
33	Hongjiang City	0	0	0	0
34	Jinzhou County	1	0	0	0
35	Tongdaozizhizhou County	2	0	0	2
36	Yuanlin County	0	0	0	0
37	Ningxiang County	0	1	0	1
38	unknown	1	0	1	1
39	unknown	1	1	1	0
Total		140	98	24	122

## Discussion

Sensitive and accurate assays are vital for efficient management of the spread of HLB-associated “*Ca*. *Liberibacter spp*”. Although HLB has been known for more than a century, “*Ca*. *Liberibacter spp”* species were identified to be associated with HLB in the 1970s [37]. “*Ca*. *Liberibacter spp*” could not be cultured, making it impossible to use traditional bacteriological methods to diagnose HLB[10]. HLB -infected trees show a lack of specific symptoms or are asymptomatic during the incubation period, and visual detection is not efficient for diagnosis of HLB. Currently, many methods based on PCR have been used to detect HLB infection, such as conventional PCR [3, 11, 12, 38], SSR[13], immune capture-PCR[17]. LAMP[16,39], PCR- RFLPs[28,40], droplet digital PCR[15], TaqMan qPCR[25], qPCR[11,12,41], qRT-PCR[4,23], and nested PCR[19]. Although these methods have worked well in symptomatic samples, they were subject to false negatives because HLB- associated Las may have a low titer and be unevenly distributed in citrus trees and even among cells within discrete tissues, yielding both positive and negative samples from various tissue samples originating from the same citrus tree [12, 42]. Each assay has advantages and disadvantages. Conventional PCR and SSR assays were simple but of low sensitivity and poor repeatability [14]. LAMP assays were simple but had high costs and poor repeatability [16]. The enzyme-linked immunosorbent assay (ELISA) was highly sensitive and target specific [43], but ELISA procedures were very complicated [35]. The qPCR was highly sensitive but had a high cost. These shortcomings are a great obstacle for these methods to practically diagnose HLB. Nested PCR, in which the products of the first round of PCR were diluted and used as the template for the second round of amplification, has been proved to have higher sensitivity than other molecular detection assays in diagnosing diseases [21–23]. In the present study, the sensitivity was in the order of nested PCR>qPCR> conventional PCR. All the results are consistent with the viewpoint that nested PCR had a higher sensitivity and was more suitable for the detection of Las with extremely low titers [44–46]. Nested RT-PCR has the advantage of improved sensitivity and specificity over conventional RT-PCR. The specificity is improved because two sets of primers are used for nested RT-PCR reactions. The sensitivity is increased because two rounds of PCR are performed in Nested PCR [18, 47, 48]. Ahmad [30] indicated that the efficiency of amplification affected the sensitivity of qPCR. In this study, the efficiency of amplification from qPCR was 99%. The results showed that the efficiency of amplification was not a key factor for the sensitivity of the assay.

The outer membrane protein (OMP) is vital for bacteria to maintain normal structure and function. OMPs are involved not only in exchanges with the external environment but also in interactions between plants and pathogenic bacteria. The three-dimensional structures of OMPs from Las were highly conserved, and the nucleotide sequences of *OMPs* from Las showed very high similarity and high species specificity (99%) [17, 29, 30]. OMPs have been used as target genes in the detection HLB bacterial assays [27– 29]. However, previous literatures indicated that *OMPs* were highly variable among different geographical isolates and were not suitable for the identification for Las [30]. OMPs were often used to produce antigens and to assess the variation among different geographical isolates. In this study, we describe a new HLB diagnosis method using nested PCR to amplify the conserved region of *BamA* that encodes the outer membrane protein (OMP) assembly factor and represents a single copy in the Las genome. The method greatly enhanced the sensitivity up to 10 times compared to qPCR and 100 times compared to conventional PCR in the detection of Las.

In the present study, 1112 samples from 5 different citrus cultivars in 39 different counties and cities were assayed by nested PCR. The results showed that 384 samples were HLB-infected, and the highest positive detection rate was 79.7% in lopsided fruit samples, and the lowest positive detection rate was 16.3% in apical dieback samples. All the results are consistent with those of earlier studies [49, 50]. The results indicate that the nested PCR primer pairs could detect Las from various different symptomatic tissues and different locations. Therefore, the nested PCR primer pairs are fit for different citrus cultivars and different geographic regions. The set of nested-PCR primers will provide a very useful supplement to the current approaches to Las detection.

## Supporting information

S1 Table Information on contacts. (XLSX)

S1 Appendix Information on sequences and BLAST online. (DOCX)

S2 Appendix All primers against citrus and Asian citrus psyllid (ACP) sequences. (DOCX)
