# Supplementary material for: Screening nested-PCR primer for ‘Candidatus Liberibacter asiaticus’ associated with citrus Huanglongbing and application in Hunan, China
Source: PLoS One. 2019 Feb 22;14(2):e0212020. doi: 10.1371/journal.pone.0212020 (PMC6386535; doi:10.1371/journal.pone.0212020)

**Information on sequences and BLAST online**

[***Candidatus Liberibacter asiaticus***](https://blast.ncbi.nlm.nih.gov/Blast.cgi#alnHdr_1390342460) [**16S rRNA**](https://blast.ncbi.nlm.nih.gov/Blast.cgi#alnHdr_220684065)

TAGGGTAATCCTTAACCCTTTTTTTATCTACGGGGATAACGCATGGAAACGTGTGCTAATACCGTATACGCCCTATTGGGGGAAAGATTTTATTGGAGAGAGATGAGCCTGCGTTGGATTAGCTAGTTGGTAGGGTAAGAGCCTACCAAGGCTACGATCTATAGCTGGTCTGAGAGGACGATCAGCCACACTGGGACTGAGACACGGCCCAGACTCCTACGGGAGGCAGCAGTGGGGAATATTGGACAATGGGGGCAACCCTGATCCAGCCATGCCGCGTGAGTGAAGAAGGCCTTAGGGTTGTAAAGCTCTTTCGCCGGAGAAGATAATGACGGTATTCGGAGAAGAAGCCCCGGCTAACTTCGTGCCAGCAGCCGCGGTAATACGAAGGGGGCGAGCGTTGTTCGGAATAACTGGGCGTAAAGGGCGCGTAGGCGGGCGATTAAGTTAGAGGTGAAATCCCAGGGCTCAACCTTGGAACTGCCTTTAATACTGGTTGTCTAGAGTTTAGGAGAGGTGAGTGGAATTCCGAGTGTAGAGGTGAAATTCGTAGATATTCGGAGGAACACCGGTGGCGAAGGCGGCTCACTGGCCTGATACTGACGCTGAGGCGCGAAAGCGTGGGGAGCAAACAGGATTAGATACCCTGGTAGTCCACGCCGTAAACGATGAGTGCTAGCTGTTGGGTGGTTTACCATTCAGTGGCGCAGCTAACGCATTAAGCACTCCGCCTGGGGAGTACGGTCGCAAGATTAAACTCAAAGGAATTGACGGGGGCCCGCACAAGCGGTGGAGCATGTGGTTTAATTCGATGCAACGCGCAGAACCTTACCAGCCCTTGACATGTATAGGGACGATATCAGAGATGGTATTTTCTTTTCGGGAGACCTTTTACACAGGTGCTGCATGGCTGTCGTCAGCTCGTGTCGTGAGATGTTGGGTTAAAGTCCGCAACTAGCGCAACCCCTGCCCTCTAGTTGCCATCAAGTTTAAGGTTTTTACCTAGATGGTTGGGTACTTTATAAGGGACCTGCCCGGTGATAATCCTGAAGGAAAGTGTGTG


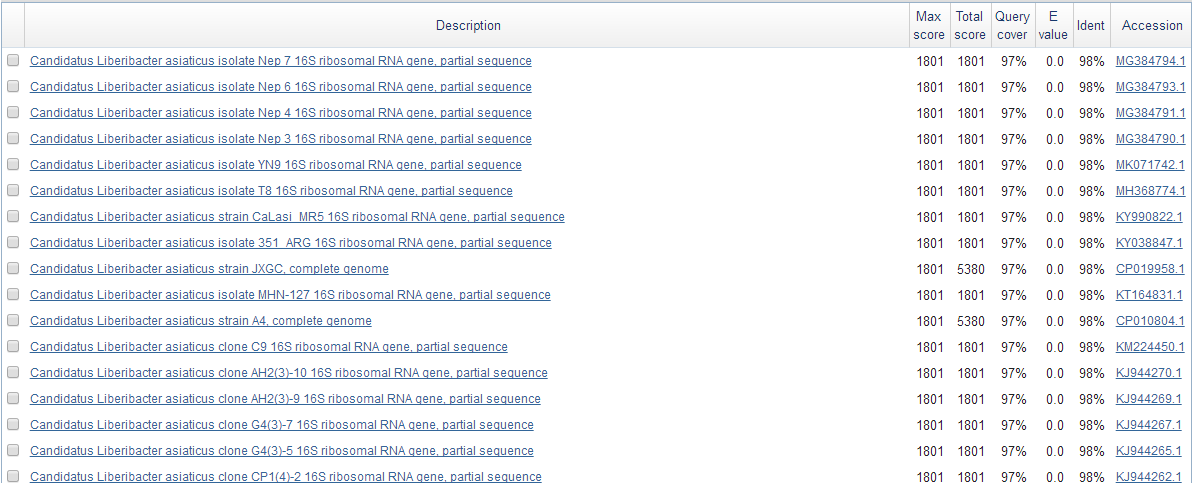


[***Liberibacter africanus*** **16S rRNA**](https://blast.ncbi.nlm.nih.gov/Blast.cgi#alnHdr_438690)

TGAGCCTGCGTTGGATTAGCTAGTTGGTAGGGTAAAGGCCTACCAAGGCTACGATCTATAGCTGGTCTGAGAGGACGATCAGCCACACTGGGACTGAGACACGGCCCAGACTCCTACGGGAGGCAGCAGTGGGGAATATTGGACAATGGGGGCAACCCGAATCCAGCCATGCCGCGTGAGTGAAGAAGGCCTTAGGGTTGTAAAGCTCTTACGCCGGAGAAGATAATGACGGTATTCGGAAAGAAGCCCCGGCTAACTTCGTGCCAGCAGCGAGCGTTGTTCGGAATAACTGGGCGTAAAGGGCGCGTAGGCGGCGATTAAGTTAGAGGTGAAATCCCAGGCTCAACCTTGGAACTGCCTTTAATACTGATTGTCTAGAGTTCAGGAGAGGTGAGTGGAATTCCGAGTGTAGAGGTGAAATTCGTAGATATTCGGAGGAACACCGGTGGCGAAGGCGGCTCACTGGCCTGATACTGACGCTGAGGCGCGAAAGCGTGGGGAGCAAACAGGATTAGATACCCTGGTAGTCCACGCTGTAAACGATGAGTGCTAGCTGTTGGGTGGTTTACCATTCAGTGGCGCACGTAACCATTAAGCACTCCGCCTGGGGAGTACGGTCGCAAGATTAAAACTCAAAGGAATTGnCGG

GGGCCCGCACAAGCGTGGAGCATGTGGTTTAATTCGATGCAACGCGCAGAACCTTACCAG CCCTTGACATATGTTGGACGATATCAGAGATGATATTTTCTTTCGGAGACTTTCATACAGGTG

CTGCATGGCTGTCGTCAGCTCGTGTCGTGAGATGTTGGGTTAAGTCCCGCAACGAGCGCAA

CCCCTACCTCTAGTTGCCATCAAGTTTAGATTTTATCTAGATGTTGGGTACTTTATAGGGACTG

CCGGTGATAAGCCGGAGGAAGGTGGGGATGACGTCAAGTCCTCATGCGCCTTATGGGCTGG

GCTACACACGTGCTACAATGGTGGTTACAATGGGTTGCGAAGTCGCGAGGCGGAGCTAATCC

CCAAAGTCCATCTCAGTTCnGATTGCACTCTGCAACTCGAGTGCATGAAGTTGGAATCGCTAG

TAATCGCGGATCAGCATGCCGCGGTGAATACGTTCTCGGGCCTTGTACACACCGC


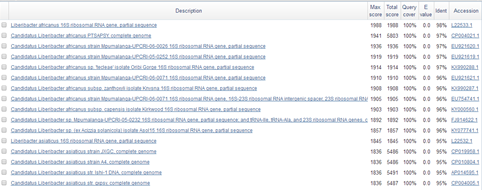


[***Candidatus Liberibacter americanus***](https://blast.ncbi.nlm.nih.gov/Blast.cgi#alnHdr_557715458) [**16S rRNA**](https://blast.ncbi.nlm.nih.gov/Blast.cgi#alnHdr_220684065)

TGTCACCACCATTGTAGCACGTGTGTAGCCCAGCCCATAAGGGCCATGAGGACTTGACGT CATCCCCACCTTCCTCCGGCTTATCACCGGCAGTCCCTATAAAGTTCCCAACTTAATGATGG CAAATATAGGCAGGGGTTGCGCTCGTTGCGGGACTTAACCCAACATCTCACGACACGAGC CTGACGACAGCCATGCAGCACCTGTGTGTGGGTCTCCGAAAAGAAAATATCATCTCTGATA TCGTCCCACCATGTCAAGGGCTGGTAAGGTTCTGCGCGTTGCATCGAATTAAACCACATGC

TCCACCGCTTGTGCGGGCCCCCGTCAATTCCTTTGAGTTTTAATCTTGCGACCGTACTCCCC AGGCGGAGTGCTTAACGCGTTAGCTGCGCCACTGAATGGTAAACCACCCAACAGCTAGCA CTCATCGTTTACAGCGTGGACTACCAGGGTATCTAATCCTGTTTGCTCCCCACGCTTTCGTG CCTCAGCGTCAGTATCAGGCCAGTGAGCCGCCTTCGCCACTGGTGTTCCTCCGAATATCAA CGAATTTCACCTCTCCACTCGGAATTCCGCTCACCTCTCCTGAACTCTAGACAAACAGTATT

AAAGGCAGTTCCAAGGTTGAGCCTTGGGATTTCACCTCTAACTTAATCGCCCGCCTACGCA CCCTTTACGCCCAGTTATTCCGAACAACGCTCGCCCCCTTCGTATTACCGCGGCTGCTGGCA CGAAGTTAGCCGGGGCTTCTTCTCCGGTTACCGTCATTATCTTCTCCGGCGAAAGAGCTTTA CAACCCTAAGGCCTTCTTCACTCACGCGGCATGGCTGGATCAGGGTTGCCCCCATGTCCAAT

ATTCCCCACTGCTGCCTCCCGTAGGAGTCTGGGCCGTGTCTCAGTCCCAGTGTGGCTGATCG

TCCTCTCAGACCAGCTATAGATCATCGCCTTGGTAGGCCTTTACCCCACCAACTAGCTAATCTA

ACGCGGGTTCATCTTTCTCCAATAAAATCTTTCCCTCTAAAGGCGTATACGGTATTAGCACACG TTCCGTGCGTTATCCCGTAGAAAAAGGTAGATCCCCACGCGTTACTCACCCGTCGCACCGTCT

CAAGCAAAAC


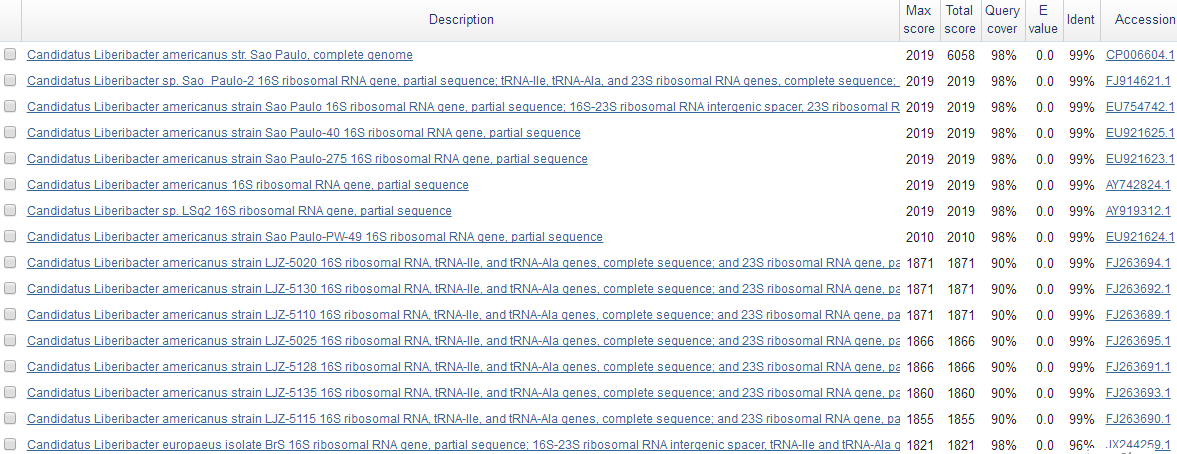


[***Candidatus Liberibacter solanacearum*** **16S rRNA**](https://blast.ncbi.nlm.nih.gov/Blast.cgi#alnHdr_1282076838)

GTTGTAAAGCTCTTTCGCCGGCTGGTCTGAGAGGACGATCAGCCACACTGGGACTGAGA

CACGGCCCAGACTCCTACGGGAGCAGCAGTGGGAATATTGGACAATGGGGGCTCCAGCC

ATGCCGCGTGAGTGAAGAAGGCCTTAGGGTTGTAAAGCTCTTTCGCCGGAGAAGATAATG

ACGGTATCCGGAGAAGAAGTCCCGGCTAACTTCGTGCCAGCAGCCGCGGTAATACGAAGG

GGGCGAGCGTTGTTCGGAATAACTGGGCGTAAAGGGCGCGTAGGCGGGTAATTAAGTTAG

GGGTGAAATCCCAAGGCTCAACCTTGGAACTGCCTTTAATACTGGTTATCTAGAGTTTAGGA

GAGGTGAGTGGAATTCCGAGTGTAGAGGTGAAATTCGCAGATATTCGGAGGAACACCAGTG

GCGAAGGCGGCTCACTGGCCTGATACTGACGCTGAGGCGCGAAAGCGTGGGGAGCAAACA

GGATTAGATACCCTGGTAGTCACGCTGTAAACGATGAGTGCTAGCTGTTGGGTGGTTTACCAT

TCAGTGGCGCAGCTAACGCATTAAGCACTCCGCCTGGGGAGTACGGTCGCAAGATTAAAACT

CAAAGGAATTGACGGGGGCCCGCACAAGCGGTGGAGCATGTGGTTTAATTCGATGCAACGC

GCAGAACCTTACCAGCCCTTGACATATAGAGGACGATATCAGAGATGGTATTTTCTTTTCGGAG

ACCTTTATACAGGTGCTGCATGGCTGTCGTCAGCTCGTGTCGTGAGATGTTGGGTTAAGTCCCG

CAACGAGCGCAACCCCTACCTCTAGTTGCCATCAAGTTTAGATTTTATCTAGATGTTGGGTACTTT

ATAGGGACTGCCGGTGATAATCCGGAGGA


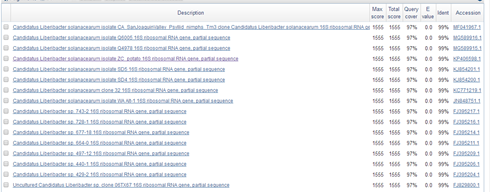


|  | [***Xanthomonas citri subsp. citri***](https://blast.ncbi.nlm.nih.gov/Blast.cgi#alnHdr_1355712843) |
| --- | --- |

CGTCGTGCGCACGATTGCGCTCGGCTCCACCGACGGCCTGAAGCGCAACCTGGTCGCCACCAACACCGAA

CGCGCCATCTCGGTGCCGGTCGGCGCCGGTACGCTGGGCCGCATCATGGACGTGCTGGGTCGCCCGATCG

ACGAAGCTGGCGACGTGCAGGCCTCGGACCATTGGGAAATCCATCGCGGCGCACCGTCGTATGAAGACCA

GTCGTCCAGCACCGAATTGCTGGAAACCGGCATCAAGGTCATCGACCTGATGTGCCCGTTCGCCAAGGGC

GGCAAGGTCGGCCTGTTCGGCGGTGCCGGCGTCGGCAAGACCGTCAACATGATGGAACTGATCAACAACA

TCGCCAAGGCGCACAGCGGCTTGTCCGTATTCGCCGGCGTGGGCGAGCGTACCCGCGAGGGCAACGACTT

CTACCACGAGATGAAGGACTCCAACGTCCTGGACAAGGTGGCGATGGTGTACGGCCAGATGAACGAGCCG

CCGGGCAACCGTCTGCGCGTGGCGCTGACCGGCCTGACCATGGCCGAGTACTTCCGCGACGAGAAAGACG

CCAGCGGCAAGGGCAAGGACGTGCTG


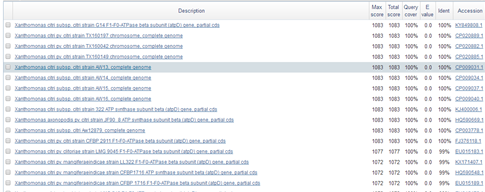


[***Spiroplasma citri***](https://blast.ncbi.nlm.nih.gov/Blast.cgi#alnHdr_1051348289)

AATTGTCAGACAACTTAGGTCATACCAACAATATTTATTTACAAATTGCACAGGATAAAAAAATTACCAA

TTATTTTGACACTGATAATGGCAAACAGTTTGAAATATGAGCGAAAGCAAATGGATACGACAATATCCGT

GGGTATAGTGCTAGTCAATTAAACAATCTTTTTGCAGATAGTAAAAATTGACAGCAACTAGCAAGTGATA

GTCAACTTGCGAGTGCGGTTGCTGATCGGGTTAAAACAAGTGGCAAGTTATCATCAACCGCACCGTTGAC

AAAAGAGCAAATGGTAGAGCAGTTAAAAACGCAAATTCCAAGCGATATCAAAATTGATAAGGTAAATACC

AACAATTATGAGAAAGATAAAGTTTCGTTTGTTTTAAACCAAAGTGAGTTTAAACCAAATGACAAAGTAA

ACATTACTGTTAAATACAATAATGCGACATCAGAACAATTTACCTTGCAAATCAAAGACAGCAATACACC

CGACAATAAAAAAGATGGTGATAGTAAATTG


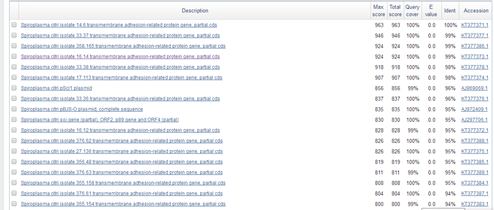


***Citrus actin***

GTATGCCACGTCGCATTCCAGACTCCACCAGAGAGAAAATACAGTGTTTGGATTGGAGGATCAATCCTTGCATCCCTCAGCACCTTCCAGCAGATGTGGATCTCAAAGGGCGAGTATGA


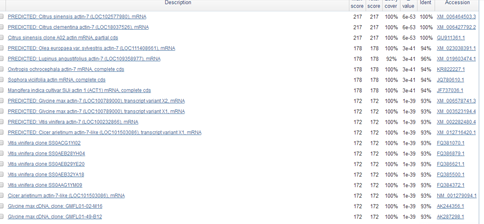


Potato actin

CTCAATCAAAGCGAGGTATCTTAACGTTGAAGTACCCGATTGAGCATGGTATTGTCAG

CAATTGGGATGATATGGAGAAGATATGGCATCATACTTTCTATAACGAGCTTCGTGTTGCACCAGAGGAG

CATCCTGTCCTCCTAACTGAAGCACCTCTTAATCCGAAAGCTAATCGTGAAAAACACAGATTATGT

TTGAGACTTTTAATACTCCAGCTATGTATGTTGCTATACAAGCTGTTCTTTCGCTGTATGCCAGTGGTCG

TACCACCGGTAAGCAAATCTTCA


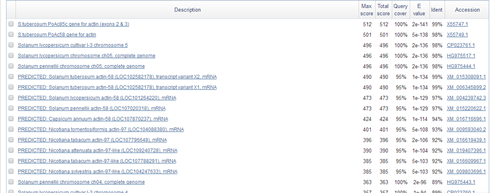


F1B1

CGGAAAGAACTCCCTGCTGCACTTGCAGACGAATAAATCTTACCTCCAATCGCATATTTCTTATCCACACGCGGACCTATACCCTTATATGCAAATCCCCTCAGATAATAATTCGAACTCACTGAGAACTGATCAAACAATTGCAAATTTTTATTGCTAGGAATGACACATCCATATCCAAATCGTAAAGAACCGACAATATCAGAATCATCTGATAGAAGATAAAAATACGATGCTCGAGATCCAATCCGATGATATTGAGAATCTCCTCCAAAACCTGCATAATCATAAGAAGATGATATCAACATGCCTTTACGTGGCACAATTGGGTTATCTAGTGTATTATAGATGATACTTTGGGAAATAGAATGGCTGCTGAATTTTCCATGTTCTATTAACGTTGTATATATCGAAGGGATCTTTTCTTTTTCTGATATAGCACCATATTGTAAAAACCTAAGATCATACTTAAAACTTGTCGATATGCTTTCAGTAATAGGAACTATCATACGTACAGCAGCAGATTCATCATTTATGTCAAGAGAGCCATCTTCAAGATGGGTTTTTTGGAGATCAAAACCCGCGGATATAGGACTCCCTAAAAAATATGGATCCTCAACACTAAAAGTATAGTTTTGTACTGCATGACGTCCAAACCCTGCCGCTAAACGAGCTCTATACCCCTGACCAAAAAAGTTATTATCATCAATATGCCCTTCTACGCCCATACCCTTATCAACTTCGTAATTAGTAGCTATACCTACAGAACCAGCGCTCAGTTGTTTTACGCTCACCCTCAGAATCACATAATCGGATACATCATTTGCTGGCAATTGCGAAATATTGACTTCAGAAAAATAACCCGTGGCCATGATACGACGCTTAGCACGCTCAATCATGGAATAATTAATTGGATCTCCCTCACTCAATTCAAGTTCACGCCGAATTACAGAATCATACGATTGATCATTCCCCTCAATTTCTATCCGCTTTACATATAACGAGACCTTGATCATCAGATACTCAATATCCACTATCCTCTTTGCAAATCTCGATATTCTCGGTCTTACCGAACAAAGCGTTCACAGAAAAGAAATATTTGAAATCTTTTCACTAGAATTCTTTATCTCCTGGTGGCTTATCCGA


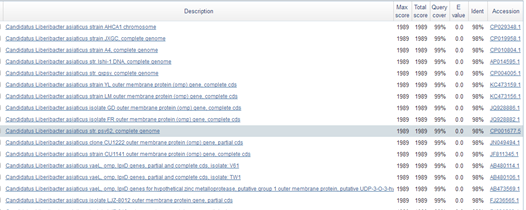


F3/B3

ATACCTGGTCTGTAGGTTAGCTACTAATTACGAAGTTGATAAGGGTATGGGCGTAGAAGGGCATATTGATGATAATAACTTTTTTGGTCAGGGGTATAGAGCTCGTTTAGCGGCAGGGTTTGGACGTCATGCAGTACAAAACTATACTTTTAGTGTTGAGGATCCATATTTTTTAGGGAGTCCTATATCCGCGGGTTTTGATCTCCAAAAAACCCATCTTGAAGATGGCTCTCTTGACATAAATGATGAATCTGCTGCTGTACGTATGATAGTTCCTATTACTGAAAGCATATCGACAAGTTTTAAGTATGATCTTAGGTTTTTACAATATGGTGCTATATCAGAAAAAGAAAAGATCCCTTCGATATATACAACGTTAATAGAACATGGAAAATTCAGCAGCCATTTAAATTTCCCAAA


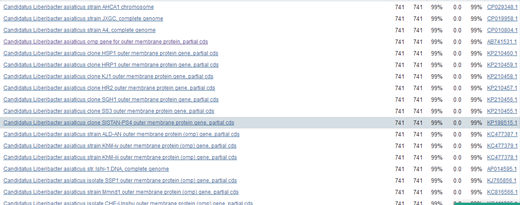

Supplement: S1 Appendix — (DOCX) [file pone.0212020.s002.docx]
