# Supplementary material for: Screening nested-PCR primer for ‘Candidatus Liberibacter asiaticus’ associated with citrus Huanglongbing and application in Hunan, China
Source: PLoS One. 2019 Feb 22;14(2):e0212020. doi: 10.1371/journal.pone.0212020 (PMC6386535; doi:10.1371/journal.pone.0212020)

**All primers against citrus and Asian citrus psyllid (ACP) sequences as following**

BLAST F1 /B1 against Citrus database


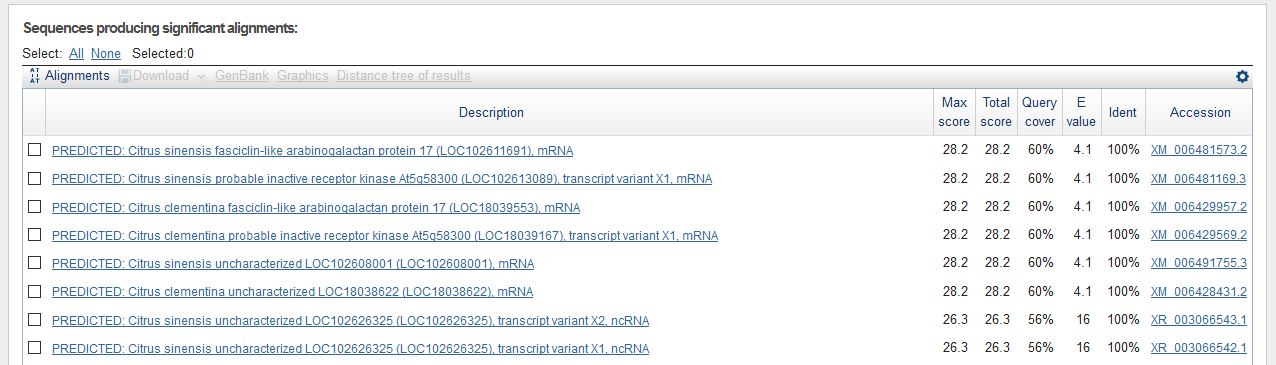


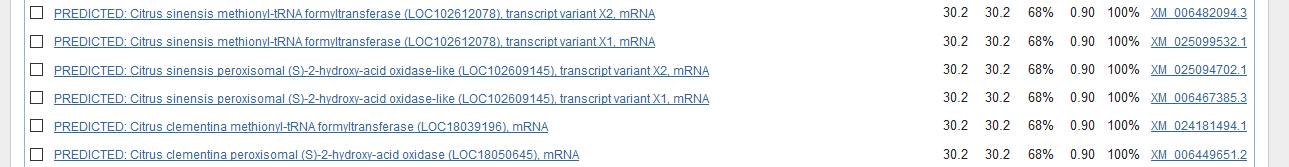


BLAST F1 /B1 against ACP database


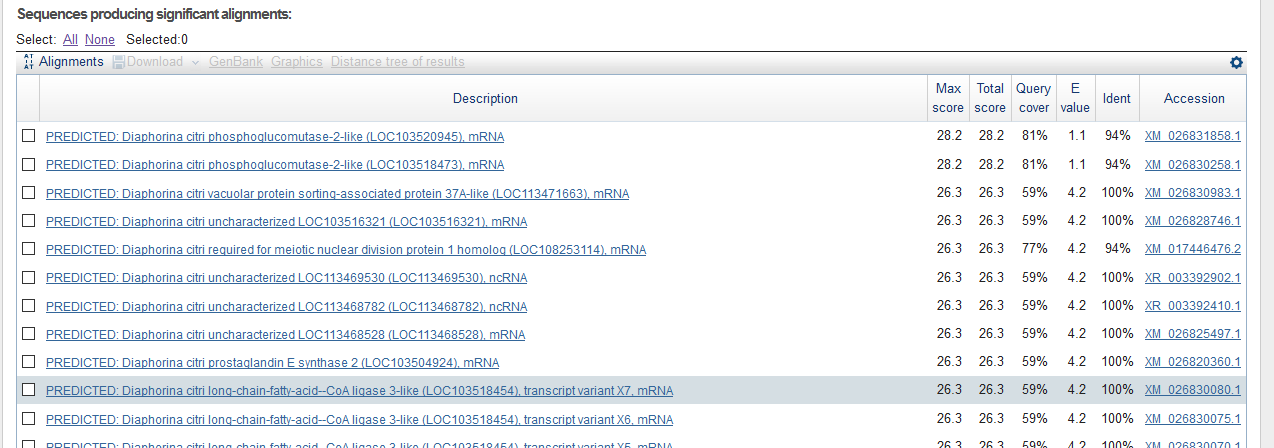


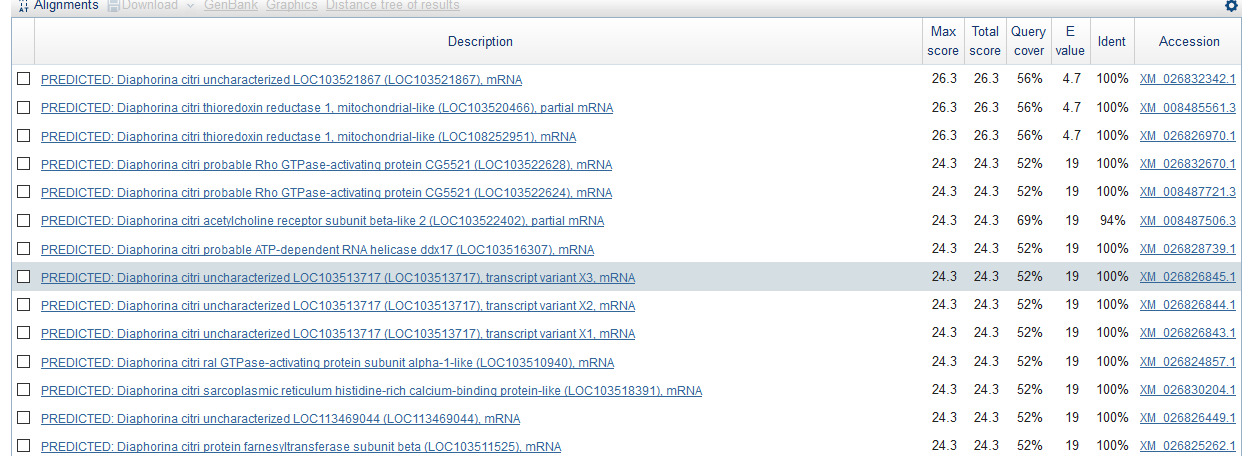


BLAST F3/B3 against Citrus database


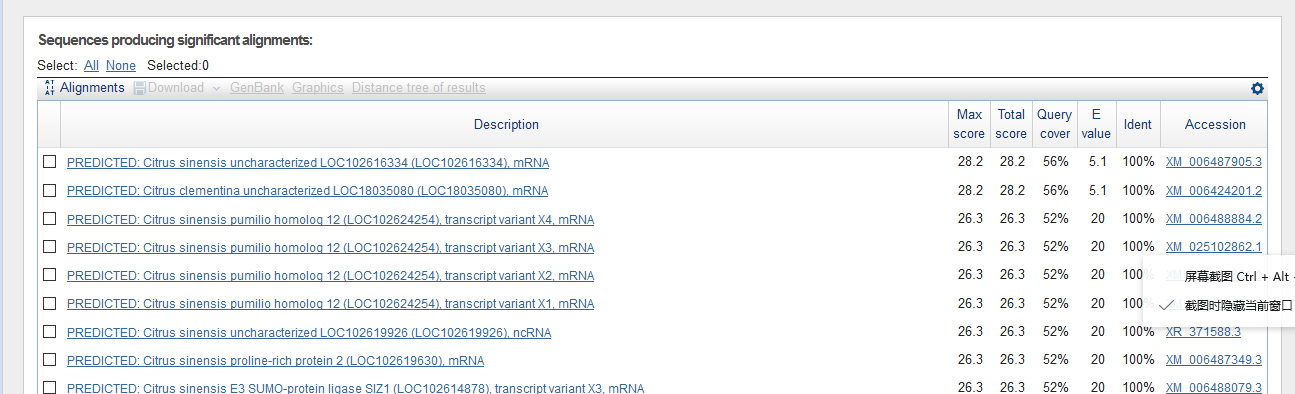


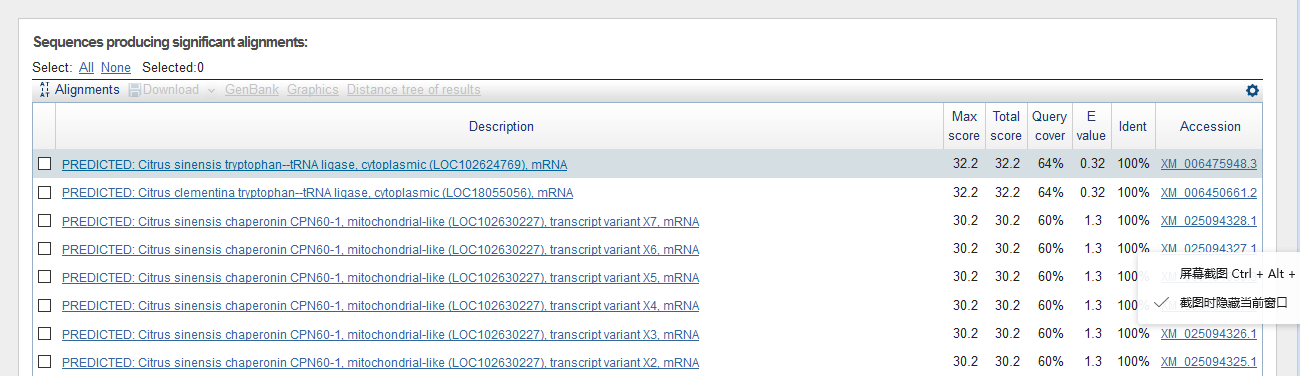


BLAST F3 /B3 against ACP database


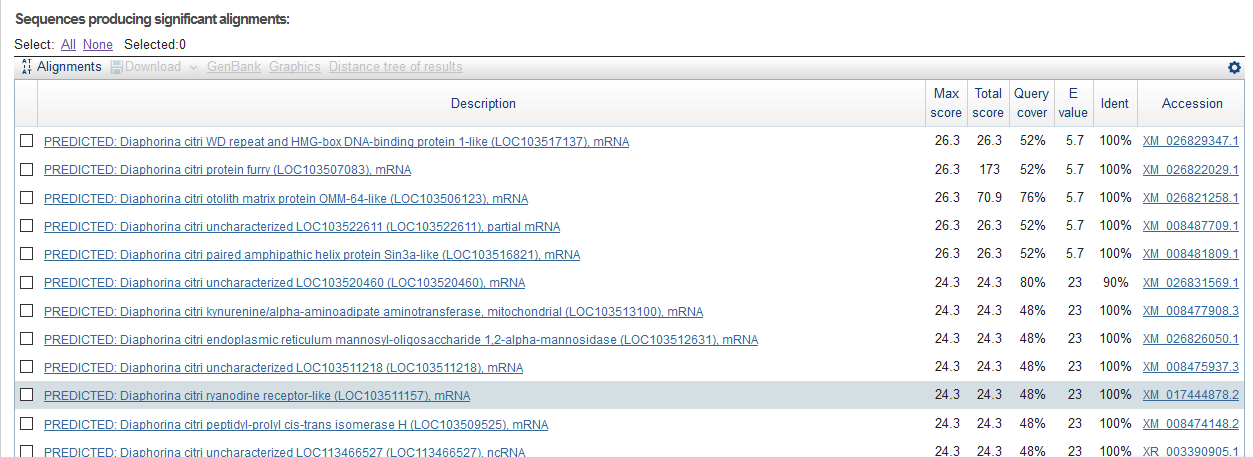


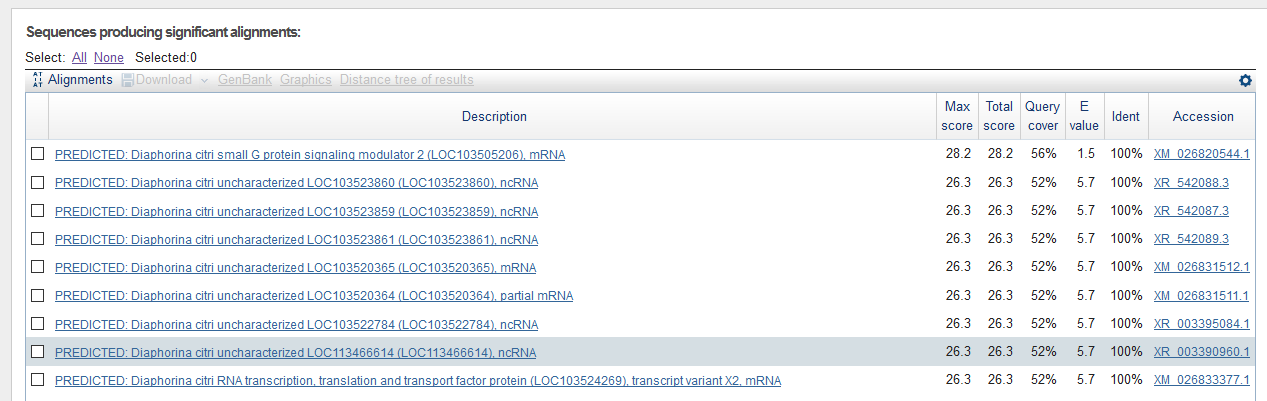


BLAST OI1 /OI2 against Citrus database


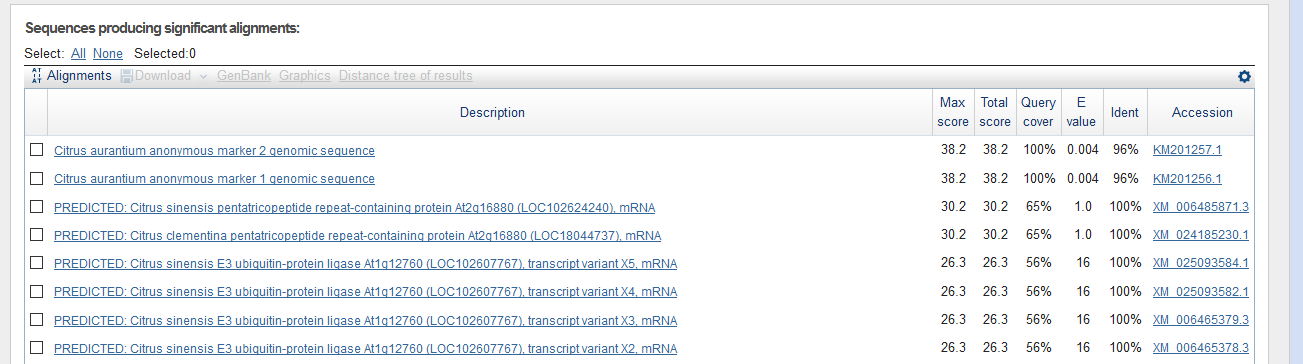


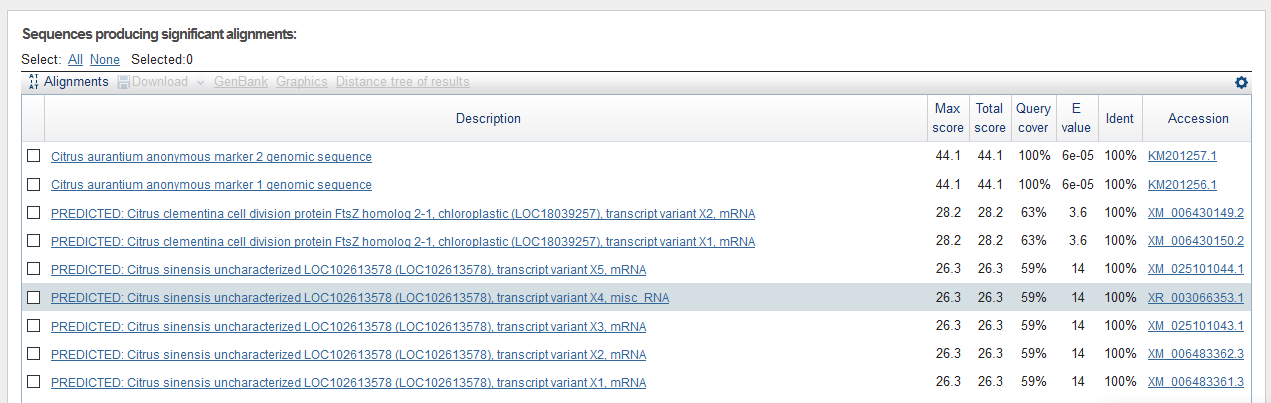


BLAST OI1 /OI2 against ACP database


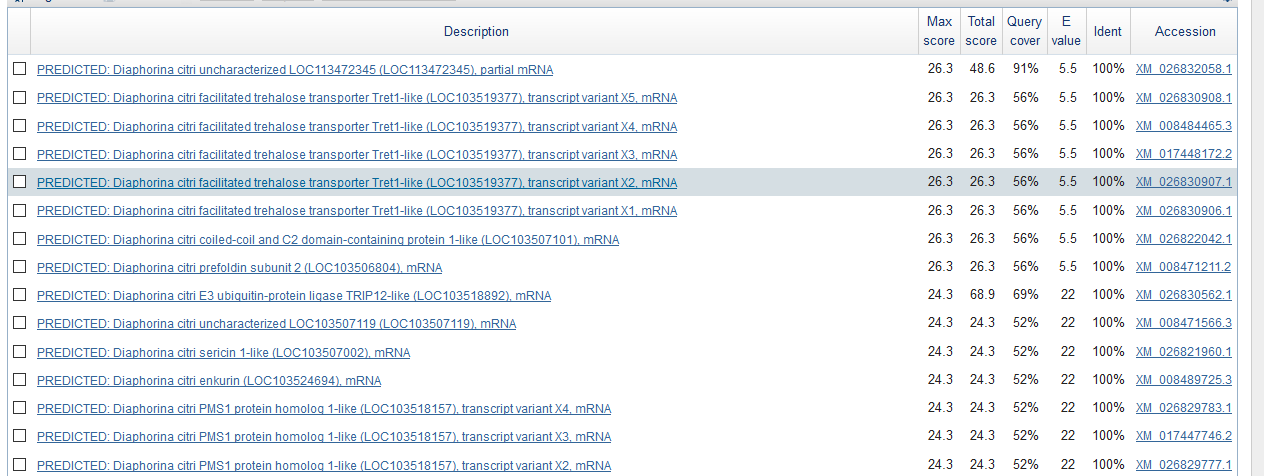


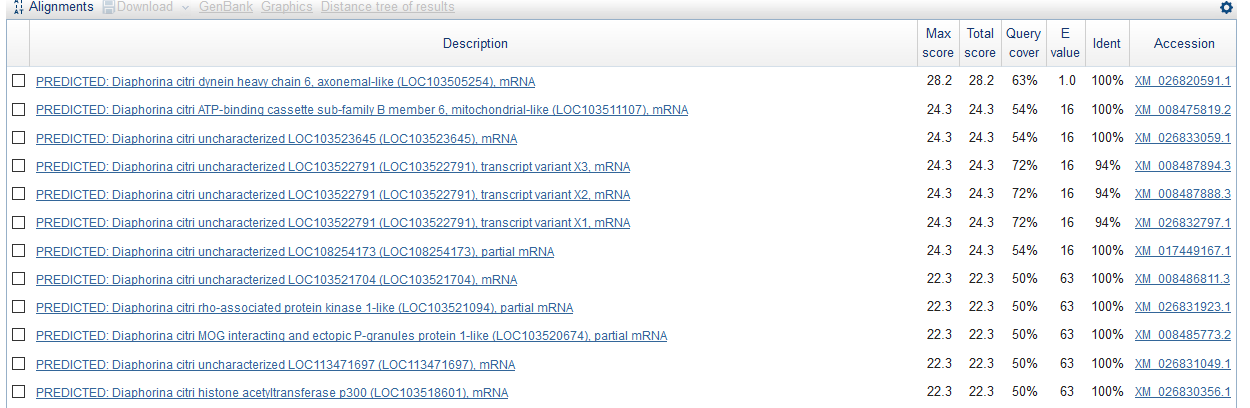


BLAST S3/S4 against Citrus database


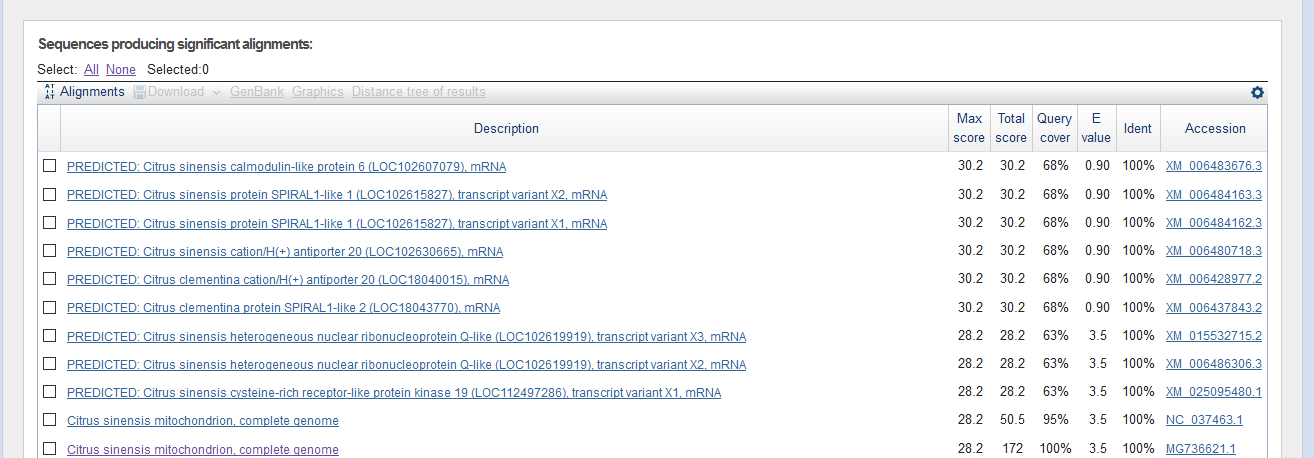


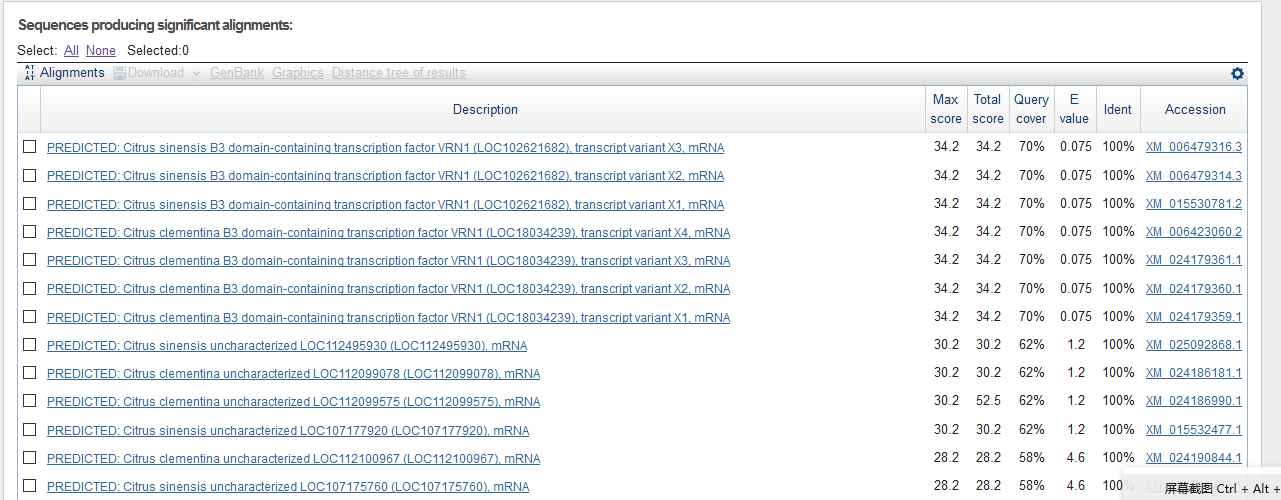


BLAST S3/S4 against ACP database


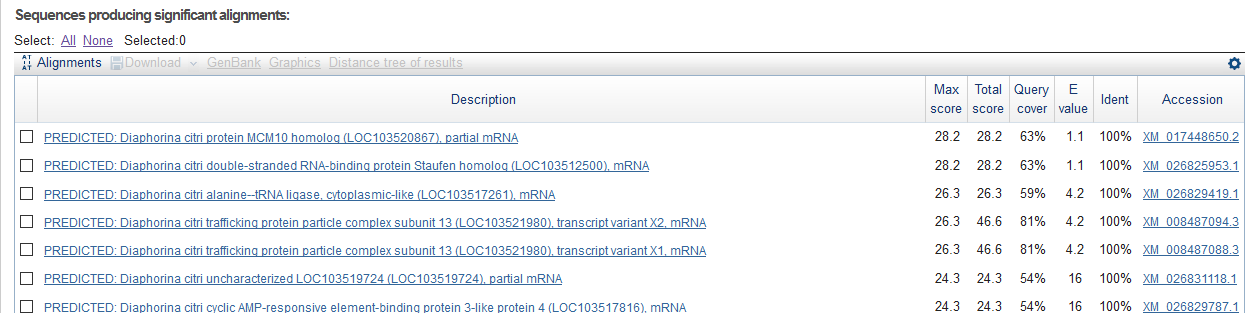


BLAST F2/B2 against Citrus database


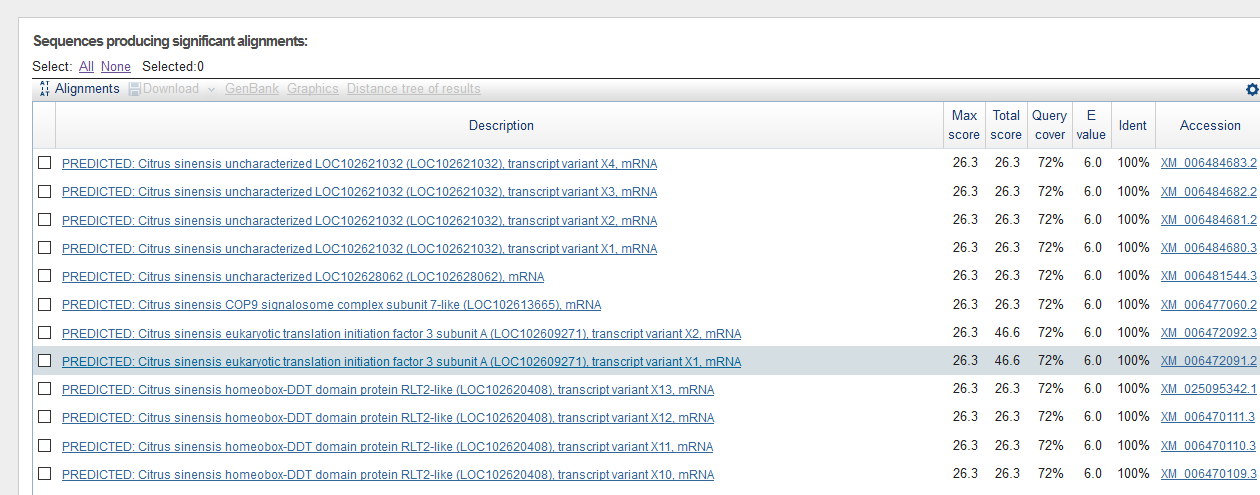


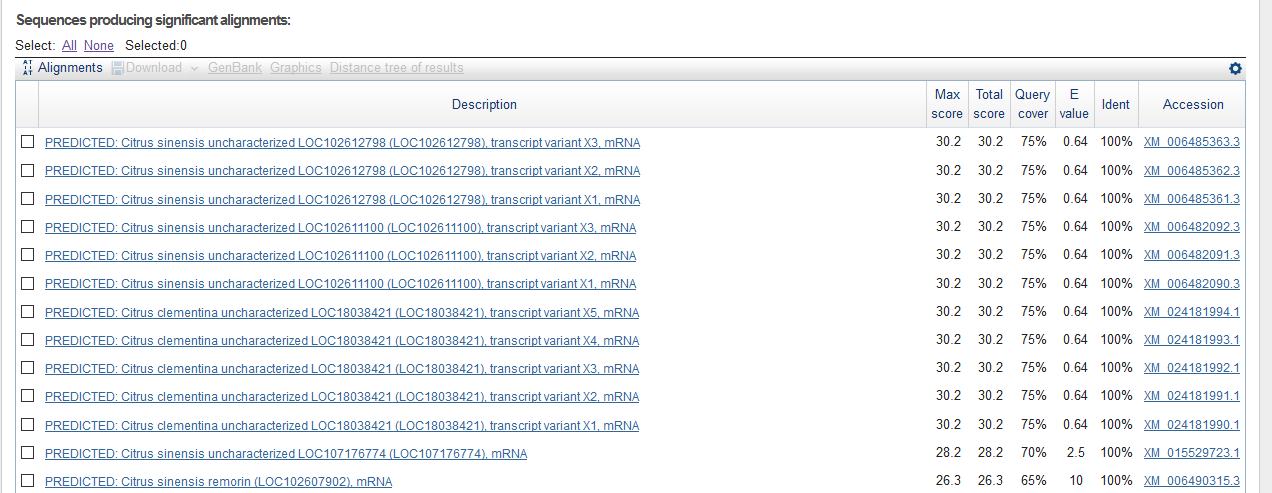


BLAST S3/S4 against ACP database


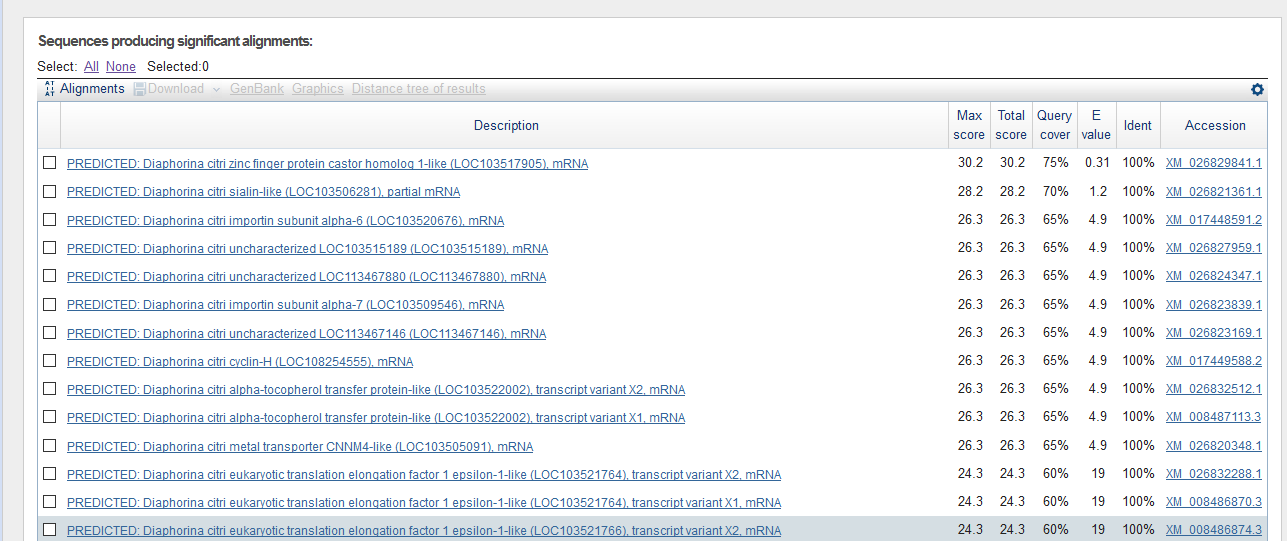


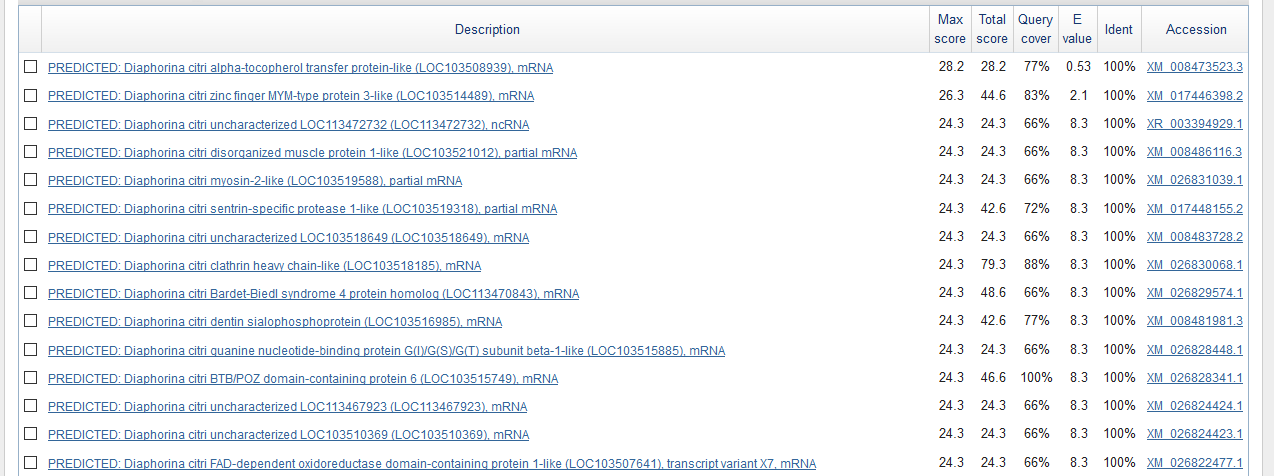

Supplement: S2 Appendix — (DOCX) [file pone.0212020.s003.docx]
